# Shh Signaling through the Primary Cilium Modulates Rat Oligodendrocyte Differentiation

**DOI:** 10.1371/journal.pone.0133567

**Published:** 2015-07-28

**Authors:** Paulina Falcón-Urrutia, Carlos M. Carrasco, Pablo Lois, Veronica Palma, Alejandro D. Roth

**Affiliations:** 1 Department of Biology, Faculty of Science, Universidad de Chile, Santiago, Chile; 2 FONDAP Center for Genome Regulation, Santiago, Chile; Rutgers University, UNITED STATES

## Abstract

Primary Cilia (PC) are a very likely place for signal integration where multiple signaling pathways converge. Two major signaling pathways clearly shown to signal through the PC, Sonic Hedgehog (Shh) and PDGF-Rα, are particularly important for the proliferation and differentiation of oligodendrocytes, suggesting that their interaction occurs in or around this organelle. We identified PC in rat oligodendrocyte precursor cells (OPCs) and found that, while easily detectable in early OPCs, PC are lost as these cells progress to terminal differentiation. We confirmed the interaction between these pathways, as cyclopamine inhibition of Hedgehog function impairs both PDGF-mediated OPC proliferation and Shh-dependent cell branching. However, we failed to detect PDGF-Rα localization into the PC. Remarkably, ciliobrevin-mediated disruption of PC and reduction of OPC process extension was counteracted by recombinant Shh treatment, while PDGF had no effect. Therefore, while PDGF-Rα-dependent OPC proliferation and survival most probably does not initiate at the PC, still the integrity of this organelle and cilium-centered pathway is necessary for OPC survival and differentiation.

## Introduction

Primary cilia (PC) are single non-motile microtubule based cell extensions that protrude from the cell body. While initially considered superfluous and thought only to play a passive role in cell proliferation through maternal centriole sequestration [1], over the last few years their role in signal interaction and integration has become increasingly recognized [2]. In particular, PC play a central role in Sonic Hedgehog (Shh) signaling, where enrichment of the effector protein Smoothened (Smo) at the surface of the PC is inhibited by the membrane receptor Patched 1 (Ptc1). Upon binding of Shh to Ptc1, PTCH1 unleashes Smoothened (SMO) from its inhibition and active Smo concentrates at the PC where it induces the activation and nuclear translocation of the Gli transcription factors [3]. While PC have been shown to play multiple roles in Central Nervous System (CNS) function and development [4–6], their participation in oligodendrocyte differentiation remains to be determined.

Oligodendrocytes (OLG) and Schwann cells ensheath and myelinate axons in tetrapods. While these cells differ in tissue localization (CNS vs. PNS), origin (ventral floor plate vs. neural crest), organization of their interaction with axons (myelination of multiple axons vs. single axon) and response to injury (reviewed in [7]); both lineages are dependent on Hedgehog signaling during embryonic development, and in the case of OLGs, Shh promotes OPC recruitment and lesion remyelinatation in a model of localized lysolecithin demyelination [8]. Still, the presence of PC to date has only been demonstrated for Schwann cells [9]. On the other hand, while OLG proliferation in response to PDGF is exploited in isolation protocols [10], and PDGF-Rα is one of the earliest markers of the OLG lineage (see [11] for review, and more recent imaging in [12]), the localization of PDGF-Rα to a PC in Oligodendrocytes has yet to be reported even though it is one of the best characterized tyrosine kinase receptors that signals through this structure in other cell types [13, 14].

Considering that two of the major pathways required for OLG proliferation, migration and differentiation [15], have been linked to the PC, it is uncanny that this structure has yet to be identified in the oligodendrocyte lineage, particularly as it could act as a signaling compartment mediating the cross-talk between PDGF-Rα and Shh [16].

Here, we report the presence of PC during rat OLG maturation as evidenced by antibodies directed against four characteristic PC markers (γ-tubulin, glutamylated tubulin, acetylated-tubulin, and ADP-ribosylation factor-like 13B [Arl-13B]) that mark structures consistent with the expected location for this organelle. We noted that as oligodendrocyte differentiation progresses, PC markers became more difficult to detect, correlating with a decline in OPC responsiveness to Shh. In addition, the crosstalk between Shh and PDGF pathways was evident, as cyclopamine inactivation of Shh signaling inhibited both PDGF-dependent cell proliferation and Shh-induced cell branching and differentiation. Furthermore, consistent with the role of PC and Shh in early OPC maturation, cyclopamine inhibited oligodendrocyte membrane sheet formation. Still, we were unable to detect PDGF-Rα distributing into the PC, suggesting that pathway interaction occurs either at the base of the PC or downstream of these receptors. Curiously, when assaying the effect of ciliobrevin, a specific inhibitor of cytoplasmic dynein, on oligodendrocyte PC, we noted that its effect on PC morphology was countered by Shh treatment, suggesting that this pathway is not only dependent on the PC but also partially capable of rescuing this organelle from drug-induced loss.

## Results

### Primary Cilium markers localize to single rod-shaped surface structures in cultured rat oligodendrocytes

**Fig 1:**
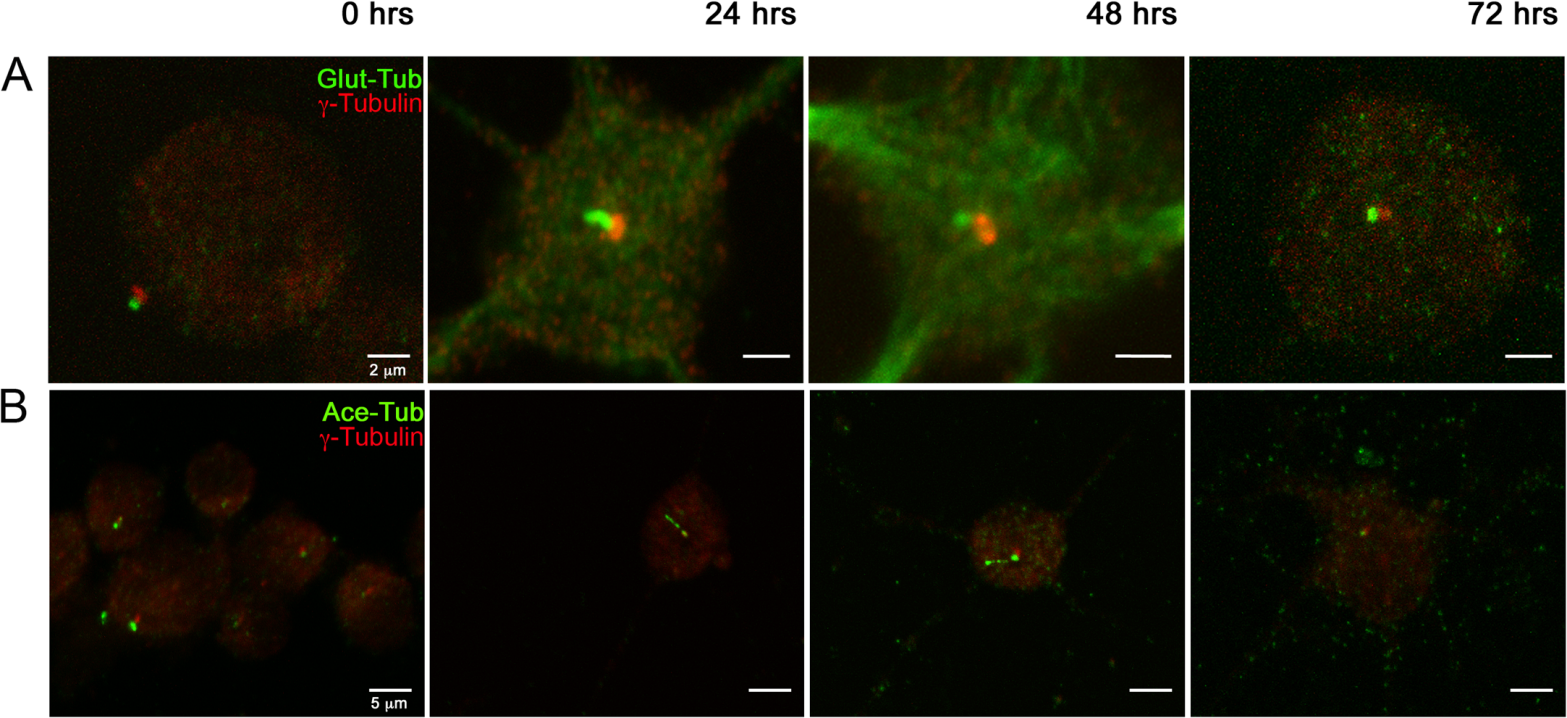
Differentiating Oligodendrocytes present PC. PC markers coincide with γ-tubulin staining on single rod-shaped structures on the surface of rat differentiating OPCs. Specific PC markers; (A) Glutamylated tubulin; (B) Acetylated Tubulin; (in green) were detected by immunofluorescence on the surface of rat oligodendrocytes 2 hrs after plating (0 hrs in differentiation medium) and for the following 24, 48 and 72 hrs. γ-tubulin staining is shown in red in all pictures. Bars: 2 μm in A and 5 μm in B.

Purified OPCs were plated on poly-lysine covered plates in DMEM-Hepes +10% FBS and allowed to attach for 2 hrs before the cell culture medium was changed to differentiation medium (designating this moment as differentiation time 0 hrs for all subsequent experiments). Cell cultures were fixed at 0, 24, 48 and 72 hrs and stained with specific antibodies directed against three well-characterized PC markers. As indicated in Fig 1, immunodetection of glutamylated tubulin (Glut-Tub, Fig 1A) and acetylated-tubulin (Ace-Tub, Fig 1B) show solitary rod-like structures that project 1 μm away from the OPC cell body which, when assayed by double immunofluorescence, display also γ-tubulin staining at their cell-proximal end (Fig 1, merged images and projection in S1 Video). These patterns are consistent with the described localization of Glut-Tub and Ace-Tub to the distal cilliary axonema, while γ-tubulin localized to the PC basal body. Given that the accumulation of Smo in the PC is one of the earliest hallmarks of canonical Shh pathway activation, we reasoned that the functionality of this organelle could be evaluated by over-expressing a constitutively active SmoA1-Flag construct that has been shown to preferentially localize to the PC in a manner similar to wild type SMO after Shh mediated inactivation of PTC1 [17]. As shown in S1 Fig, transfected cells presented an enrichment of Flag-expressing proteins in areas apposing γ-tubulin staining, a pattern reminiscent of activated SMO.

**Fig 2:**
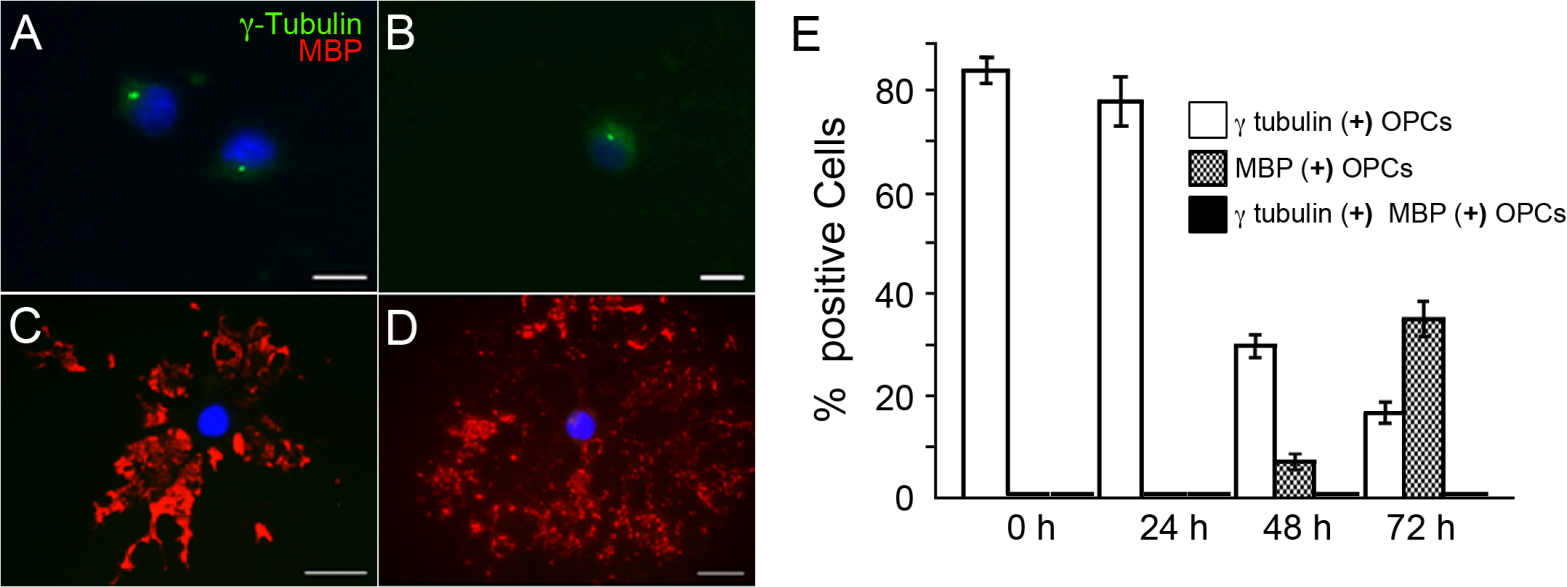
γ-Tubulin positive axonemes are undetectable in OPCs expressing the differentiation marker MBP. Isolated rat Oligodendrocytes were cultured in differentiation medium over 72 hrs. At set time-points (A: 0 hrs; B: 24 hrs; C: 48 hrs; D: 72 hrs) cultures were fixed and stained for γ-tubulin (A, B) and MBP (C, D). DAPI was used for nuclei stain. Bars: 2 μm (E). Random fields were photographed in the 488 nm (γ-tubulin) and 543 nm (MBP) channels and cells were scored double blind. The graph represents the mean percentage of marker (+) cells ± s.d. of three independent experiments. *p< 0,01 (Kruskal Wallis test).

PC markers were detected through multiple OPC differentiation stages (Fig 1) but seem more prominent and easier to distinguish in sparsely branched cells and at shorter differentiation times (Fig 1, compare at 0, 24, 48 and 72 hrs). The marker association to cells with morphological characteristics of undifferentiated OPCs and/or immature OLGs contrasted with reduced PC staining in cells with abundant branching processes, characteristic of differentiated OLGs. We confirmed this observation in OPCs fixed at multiple differentiation times and stained for γ-tubulin and Myelin Basic Protein (MBP), a classic marker of OLG terminal differentiation [11]. As expected, MBP increased profusely and, after 72 hrs, was present in the fused processes that make up the characteristic membrane sheets of *in vitro* differentiated OLGs [18] while γ-tubulin staining was evident in MBP negative cells, particularly at early differentiation times (Fig 2A–2D). The presence of either marker was scored in double blind fashion in 300 cells per treatment, confirming these observations (Fig 2E). It is remarkable that MBP positive cells did not present γ-tubulin staining; which we expected to mark at least the presence of centriolar axonema. Still, this observation is consistent with the work of other authors who have noted a lack of fluorescent labeling of the OLG microtubule organizing centers [19].

### OPC responsiveness to PDGF is modulated by the Shh pathway

**Fig 3:**
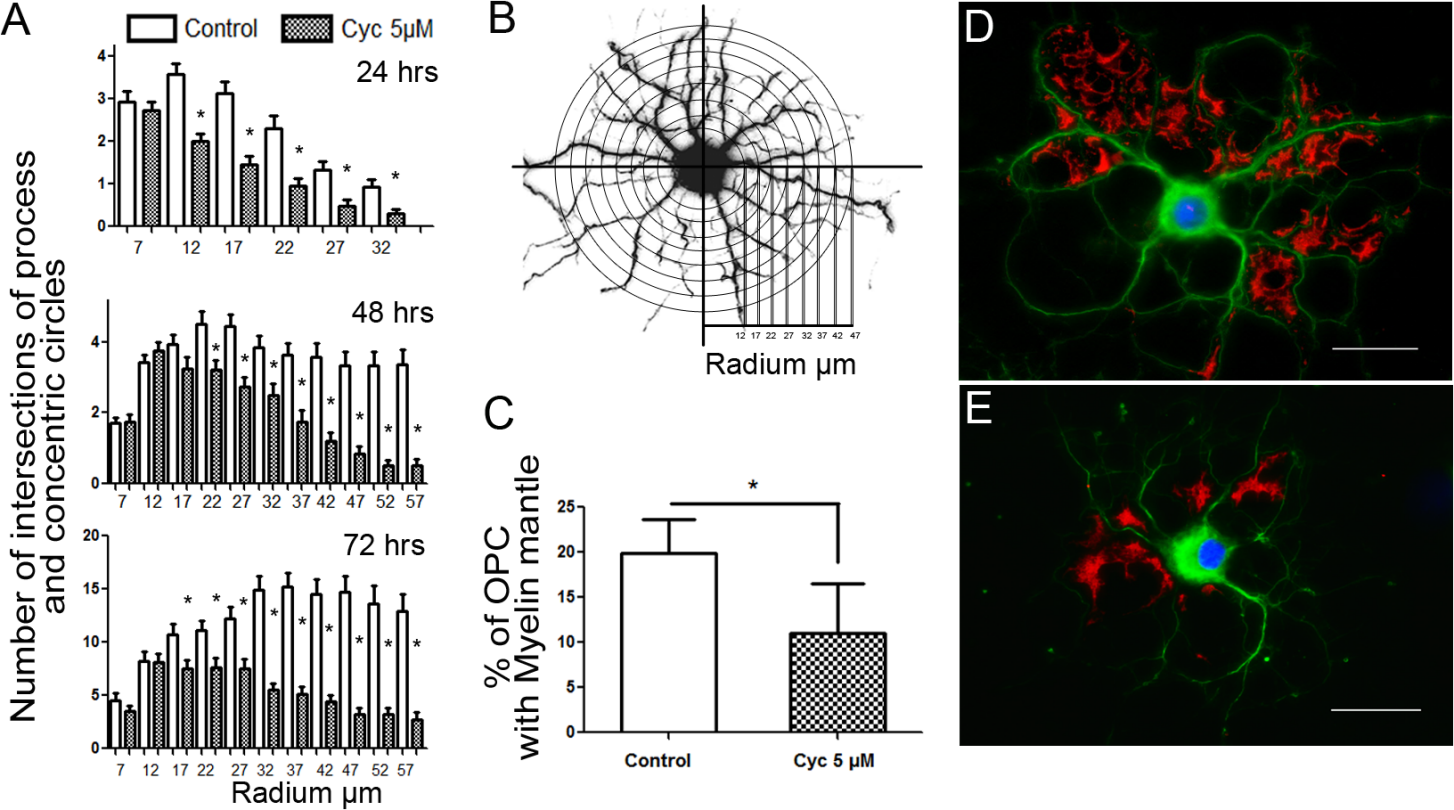
Cyclopamine, a Hedgehog pathway antagonist, inhibits *in vitro* differentiation of Oligodendrocytes. (A) Shh antagonist (Cyclopamine, 5μM) significantly inhibits OPC differentiation at 24, 48 and 72 hrs as assayed by process extension and branching evaluated through Scholl analysis. Each graph represents the distribution of cell processes crossing superimposed concentric circles marking distance from soma ± s.d. of three independent experiments. *p< 0,01 (Kruskal Wallis test). (B) Scholl analysis on color-inverted image of α-tubulin stained differentiating OPC with superimposed concentric circles marking distance from soma. (C) Cyclopamine (5μM for 72 hrs) significantly reduced MBP-rich membrane expansions. The graph represents the mean percentage of MBP+ cells ± s.d. of three independent experiments. Representative images of differentiated control and Cyclopamine-treated oligodendrocyte stained for MBP and α-tubulin (D, E). Bars: 10 μm. Cyc: Cyclopamine.

Shh plays a pivotal role in the specification and proliferation of embryonic spinal cord OPCs [20, 21, 22, 23]. We evaluated the effect of cyclopamine, a potent Hedgehog inhibitor, on *in vitro* OPC differentiation and we found that inactivation of Smo by 5 μM cyclopamine induced a time-dependent reduction in OPC process branching as determined by Scholl analysis while standard differentiation conditions induced a time-dependent increase of cell branching, reflected in both the cell length of cell processes and the number of concentric rings intersected by cell processes (see Fig 3A and 3B). After 72 hrs this resulted in a significant number of cells with the characteristic morphology of mature OLGs, presenting an increase in the number of MBP-positive membrane sheaths. Contrarily, cyclopamine treatment induced a significant delay in both process extension and branching at all assayed times, while significantly reducing the number of detectable MBP-positive membrane sheaths after 72 hrs of treatment (Fig 3D and 3E, quantified in C), confirming the blockage of OPC differentiation by this alkaloid.

**Fig 4:**
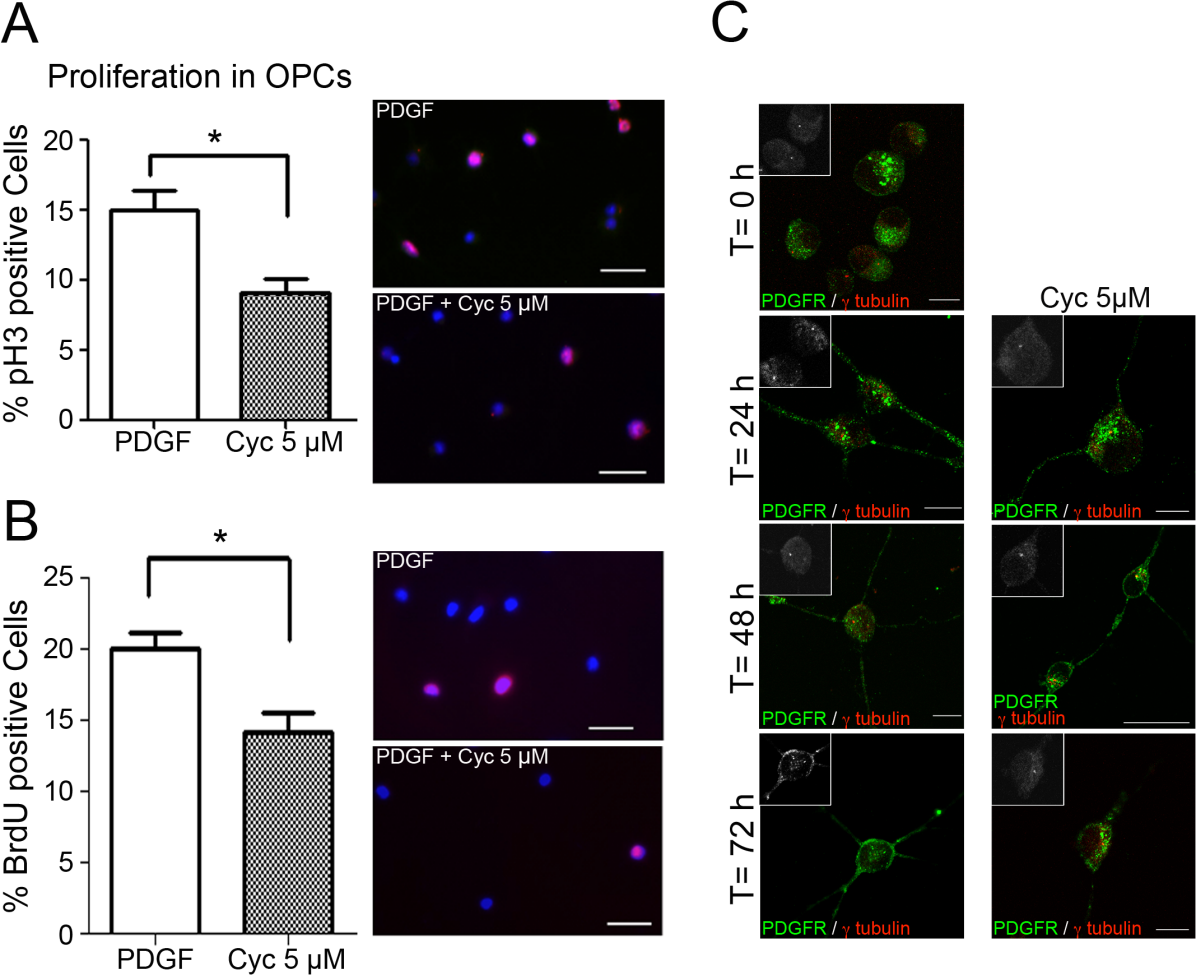
Cyclopamine inhibits PDGF-AA mediated PC proliferation, but PDGF-Rα does not localize to the PC. Isolated OPCs proliferate in response to PDGF-AA (10 ng/ ml), but in the presence of cyclopamine (5 μM) cell proliferation is inhibited as evidenced by a reduction of phosphorylated Histone-3 (pH3) (A) and BrdU incorporation (B) Bars: 10 μm. Graphs represents the mean percentage of marker (+) cells ± s.d. of three independent experiments. *p< 0,01 (Kruskal Wallis test). (C) PDGFR (green) and γ-tubulin (red and insets) localization was assessed by immunofluorescence in differentiating OPCs in the presence or absence of 5 μM cyclopamine (Cyc 5 μM). Bars: 20 μm.

Like Shh, the PDGF-Rα signaling pathway has been linked to the PC [14] and plays an indispensable role in OPC proliferation both *in vitro* and *in vivo* [15]. Still, in spite of the well documented importance of both pathways in OPC proliferation, migration and differentiation, information on possible cross-talk is scarce. Therefore, we characterized the effect of Shh-pathway inhibition on PDGF-induced OPC proliferation. For this, purified OPCs at time 0 received a proliferation medium including 10 nM PDGF-AA, either in the presence or absence of the Hedgehog inhibitor. After 24 hrs, Smo inhibition in the presence of 5 μM cyclopamine induced a significant drop in OPC proliferation, as evidenced by the significant loss of Histone-3 phosphorylation and BrDU incorporation (Fig 4A and 4B, respectively) confirming the cross-talk between these pathways in OPCs. Toxic effect by cyclopamine was ruled out by analyzing Lactate Dehydrogenase (LDH) release over 24 hrs in the presence of 1, 5, and 10 μM cyclopamine (S2 Fig). In view of these results, we considered it likely that PDGF-Rα localization would reflect the pathway interaction by coinciding within the PC. But when PDGF-Rα and γ-tubulin localization were assayed, both in the presence and absence of 5 μM cyclopamine, no significant localization of PDGF-Rα to the PC could be observed. Rather, PDGF-Rα appeared distributed in patches over all OPC membranes and extensions, as observed by other authors (for reviews see [11, 15]]. While such distribution generally results in a PDGF-Rα patch close to the base of the PC, PDGF-Rα, we did not observe a significant correlation of patches to PC, and could not detect PDGF-Rα in the PC itself (Fig 4C).

**Fig 5:**
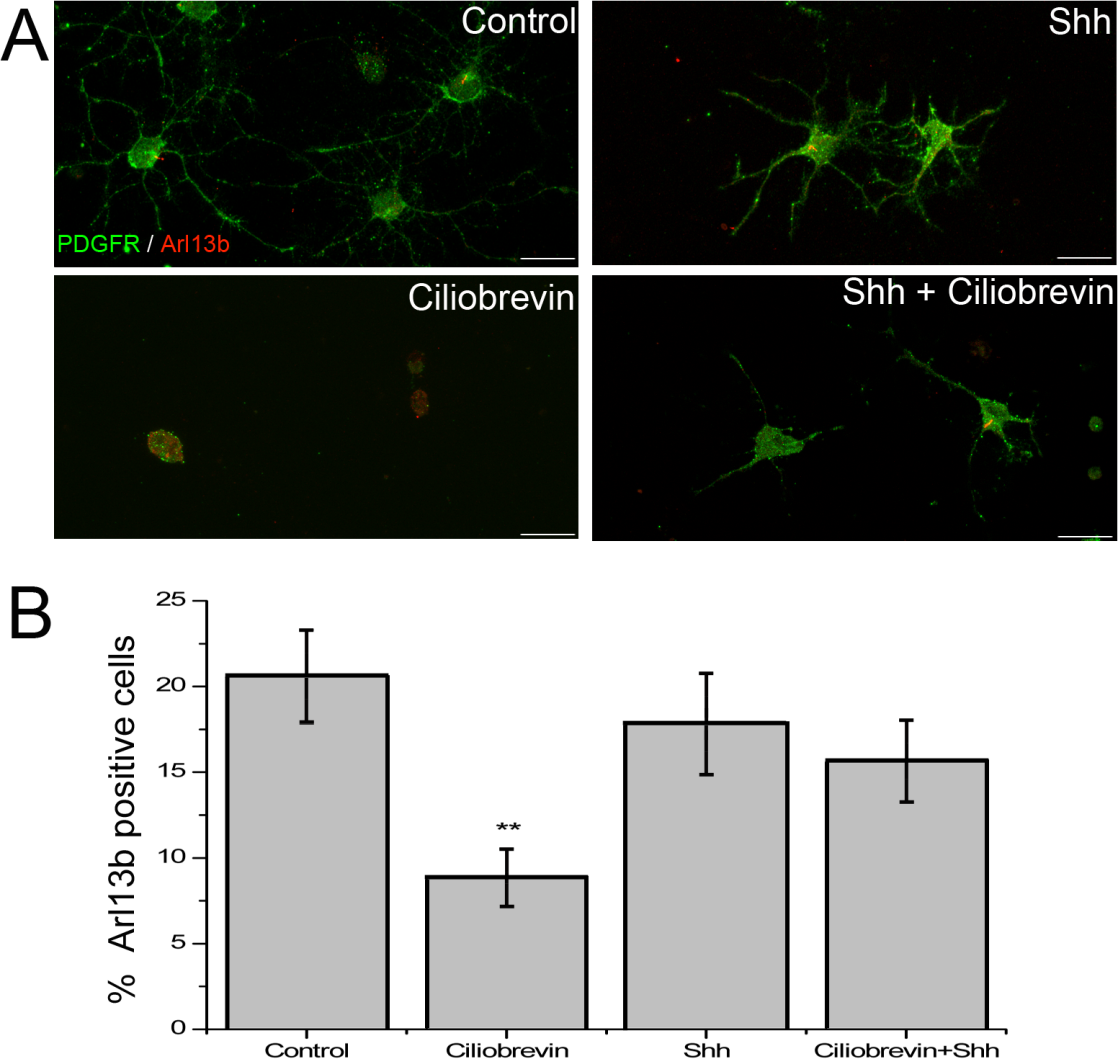
Shh rescues PC and Oligodendrocyte morphology from ciliobrevin. A. Proliferating OPCs (medium supplemented with PDGF-AA) were pretreated for 3 hrs with ciliobrevin (30 μM) or with proliferation medium, after which they received 3.3 μg/ml of recombinant Shh and were cultured for 24 hrs before assessing PDGF-Rα (green) and Arl-13B (red) localization by immunofluorescence. Shh supplementation restored PC presence and structure. Bars: 20 μm. B. Quantification of 30 random fields scored for the presence of Arl-13B (+) and counted as a percent from total Dapi (+) cells. Graph shows percent of Arl-13B positive cells ± s.e.m. *P< 0,01 (Kruskal Wallis test).

A recent report by Kim et al [3] has shown that ciliobrevin, an AAA+ ATPase inhibitor which blocks retrograde transport into the PC [24] and results in PC disassembly, also stabilizes Smo localization to the remaining PC by reducing both the rate of entry and exit into this organelle [3]. In order to determine if such stabilization could evidence a PDGF-Rα distribution into the PC, OPCs under proliferative conditions (+PDGF-AA) were pre-treated for 3 hrs with 30 μM ciliobrevin. Following this incubation, cells were subjected to 3.3 μg/ml of recombinant Shh, cultured for 24 hrs in the presence or absence of ciliobrevin; fixed and stained for Arl-13B (to reveal location of remaining PC) and PDGF-Rα. Cells treated only with Shh presented multiple processes (Fig 5A) and did not differ in the number of PC detected when compared to control cells (quantified in Fig 5B). While cells treated with ciliobrevin in the absence of Shh presented a rounded morphology and appeared to be severely damaged (see Fig 5A), there was no increase in the activity of LDH in the cell medium (S2 Fig) and no evident change in the number of cells per field (S3 Fig). Still, some Arl-13B positive puncta could be detected, but did not present morphology consistent with PC (Fig 5A). Nevertheless, even if these Arl-13B positive puncta were quantified as *bona fide* PC, there was a pronounced loss of PC in response to ciliobrevin (Fig 5B) and, more importantly, no concentration of PDGF-Rα into these organelles. In contrast, when cells where treated with Shh after the three hour ciliobrevin pre-treatment, they presented a branched morphology comparable to control cells (Fig 5A) and normally shaped Arl-13B positive PC which, when quantified, yielded a normal number of PC-yielding cells, comparable to control and Shh-only treated cells (Fig 5B). In this sense, while it would seem evident that ciliobrevin-dependent inhibition of a dynein should have caused a cell destabilizing effect and ciliary loss, it is remarkable that co-treatment with Shh would protect the OPCs and prevent PC disassembly.

## Discussion

CNS myelination by oligodendrocytes requires the emergence, migration and proliferation of OPCs, starting from their initial sites of induction (ventral, dorsal, caudal or cortical [25]) to a generalized distribution throughout the CNS. Over the course of these events OPCs are sensitive to the signals provided by the floor plate, the surrounding cells and the extracellular components [20]. Such a myriad of signaling cues must be integrated in order to change from migration to proliferation, and subsequently into postmitotic cells that initiate axonal myelination [15, 26]. Among the early signaling cues, the Shh pathway induces OPC development both from ventral [27] and cortical [28] niches and ushers in the expression of recognized differentiation markers such as PDGF-Rα, which mediates proliferation and migration of OPCs in response to PDGF-AA [29, 30]. Still, though PDGF-Rα is extensively used as a marker of OPCs [10], unambiguous localization of these receptors to PC in the CNS has only been reported for sub-ventricular zone neural stem cells and neural progenitors [31] and not for OPCs. Interestingly, while the association of Shh, PDGF and PC in oligodendrocytes has been previously suggested in a review [1], the reference cited does not show the localization of PC in these cells [32], nor have the functional implications of these interactions been explored.

Here we describe the presence of multiple PC markers (γ-tubulin [33], glutamylated tubulin [34], acetylated tubulin [33] and Arl-13B [35] in structures with size and shape consistent with PC. We must note that, although acetylated tubulin is normally used as a conventional PC marker [33], this is the first report characterizing its localization to the PC of cultured oligodendrocytes. However, this is not the first report of OPC Ace-Tub, as previous authors have shown it to be distributed throughout OPC tubulin cytoskeleton [19], where it marks the increasingly complex arborization of cell processes [36]. We traced this discrepancy to the use of paraformaldehyde versus methanol fixation, for when fixations were carried out with the latter, Ace-Tub staining resulted in well-defined PC (Fig 2) consistent with other PC markers employed. Conversely, when 4% paraformaldehyde was used for fixation, acetylated-tubulin staining was observed over most cell processes and soma (S4 Fig), which is consistent with the previously reported distribution [19, 36]. Whether these variations reflect a differential access to the overall acetylated tubulin or a difference in antibody reactivity remains to be determined and is beyond the scope of this article.

As proliferating OPCs go from migratory-bipolar cells to increasingly ramified pre-oligodendrocytes and progress to postmitotic immature oligodendrocytes, they lose PDGF-Rα expression and responsiveness to PDGF (for review see [11]]. We observed that these differentiation steps coincide with the loss of PC markers, suggesting that differentiated oligodendrocytes lose this structure as they approach terminal differentiation. While this is contrary to the notion that PC are a characteristic of mammalian cells that enter growth arrest [2], the loss of PC as cells terminally differentiate has been described in other cell types [37, 38]. Nevertheless, we were particularly surprised by the absence of γ-tubulin staining in differentiated oligodendrocytes, since we expected this epitope to have been detectable at the microtubule organizing centers. In line with our observations, lack of γ-tubulin staining in differentiated oligodendrocytes has been previously observed and described [19]. Coincidentally, a recent report on Schwann cell PC has explained a similar phenomenon of PC loss as these cells initiate myelination [9]. Further studies should discern the cellular conditions under which PC can promote or inhibit either proliferation or differentiation, keeping in mind that these functions could be context dependent, promoting proliferation in some contexts (i.e. postnatal dentate gyrus) while restraining it in others (such as the embryonic cortex).

As expected, PC are present in myelinating cell populations during their Shh responsiveness window, a time when these cells are migratory. At the same time, the importance of Shh [27] and PDGF-Rα [39] during oligodendrogenesis, coupled with their reported localization to the PC suggested that: a) a ciliar dependent-or directionalized- mechanism of PDGF-AA chemotaxis could be localized to this organelle, as described for fibroblasts [40]; and b) Shh and PDGF-Rα cross-talk could occur in the PC. In this sense, it is unlikely that PC can act as a PDGF-AA directional sensor in differentiating OPCs, as PDGF-Rα does not localize to this organelle and distributes as patches on the surface of OPCs (as shown by other authors, [12], and [11] for review). On the other hand, we observed that OPC-differentiation was particularly sensitive to the inhibition of the Shh pathway by cyclopamine, a well-characterized inhibitor of Hedgehog signaling that acts by blocking Smo activation, coinciding with Lelievre et al.[41] who showed that OPC proliferation in response to Shh and PDGF could be antagonized by pituitary adenyl activating peptide (PACAP). While these authors suggest that the effects of both pathways are independent from each other, we clearly observed that cyclopamine significantly inhibited PDGF-AA dependent OPC proliferation. This interaction, and the presence of the PC, suggest that these pathways could interact at this organelle, but as we failed to detect the presence of PDGF-Rα at the PC either in the presence or absence of cyclopamine, it seems unlikely that such interaction occurs between the receptors themselves. Still, the PC appears to be extremely important for PDGF-AA signaling, as ciliobrevin mediated disassembly of this organelle counteracts PDGF-AA induced OPC proliferation and survival, effects which were countered by adding Shh to ciliobrevin pre-treated cell cultures, rescuing both cell morphology and PC. Even though the relationship between the PC structure and ciliobrevin affects the presence and turnover of the Smo in the PC [3], there is no evidence to date showing the opposite dynamics, considering the regulation that the Hedgehog signaling is capable of exerting over the PC structure. Therefore, this evidence may account for a new cellular process regulated by Hh that might have biological implications in terms of the classical role that Hh display in different contexts. Further work should clarify the feedback mechanisms between Shh signaling and PC structural stability.

In conclusion, while PC are considered to be present in most vertebrate cells (http://www.bowserlab.org/primarycilia/cilialist.html), they are particularly enriched among cell cycle arrested cells (for review, see [2] and references therein), although a few cell types escape this generalization. Myelinating cells in particular (oligodendrocytes and Schwann cells) present multiple differences that unequivocally distinguish both lineages. Still, both present migratory behaviors in their early differentiation stages, well before they engage and ensheath axons. We find that, like Schwann cells, oligodendrocytes also present PC during their initial developmental stages and that this signaling organelle becomes undetectable as cells differentiate. A transient increase in OPCs by Shh pathway activation could explain a subsequent increase in production of oligodendrocytes derived from such expanded progenitor pools. We cannot rule out that Shh signaling is also required to determine the fate of already committed OPCs, but at this time we favor the hypothesis that Shh is acting on OPCs by promoting their proliferation. Considering the importance of OPCs in the etiology of various demyelinating diseases and the role of PDGF-AA and Shh on OPC proliferation and recruitment to demyelinating lesions [8], further work should address whether Shh signaling at the PC of oligodendrocyte precursors is implicated in cell proliferation after external insults *in vivo* and/ or as part of normal homeostasis.

## Materials and Methods

Unless specified otherwise, all chemicals were obtained from Sigma-Aldrich. Antibodies were: acetylated α-tubulin (T7451, Sigma-Aldrich, and D20G3; Cell Signaling Technology), ADP-ribosylation factor-like 13B (Arl-13B, NeuroMab clone N295B/66), Glutamylated tubulin (AB3201, Chemicon), Myelin basic protein (MBP, Aves Labs), γ-tubulin (T6557, Sigma-Aldrich), Histone-3P (06–570, Millipore), BrdU (M0744, DAKO) and PDGF-Rα (SC-338, Santa Cruz Biotech).

This study was carried out strictly in accordance with animal use guidelines of the National Fund for Science and Technology (FONDECYT) and with the Guide for the Care and Use of Laboratory Animals issued by the Institute for Laboratory Animal Research of the National Research Council (USA; National Academies Press, 2011). All procedures were approved by the Ethics Committee of the Faculty of Sciences, the University of Chile (certificates issued on July 31st, 2012 and October 4th, 2013) and were reviewed by the Bioethics Committee of the National Fund for Science and Technology (FONDECYT). Animals were neonatal (P1) Sprague Dawley rats obtained from the central animal housing facilities at the Catholic University of Chile. Suffering of animals was kept to a minimum and no procedures inflicting pain were performed.

### Cell culture and transient transfection

OPCs were purified by differential shaking of mixed glial cell cultures [42] established from neonatal (P1) rat cortices. Briefly, P1 animals were sacrificed by decapitation, cortices dissected, placed in ice-cold L15 Medium (LifeTechnologies, USA) and chopped with a scalpel. Tissue was incubated at 37°C for 10’ with 1X trypsin (0.25% LifeTechnologies) supplemented with 200 Units of DNAse (Sigma-Aldrich). Trypsin was inhibited by dilution in L-15 + 10% FBS and cell mixture was spun for 5 minutes at 1000 rpm in a clinical centrifuge. Ensuing pellet was resuspended in DMEM-HEPES+10% FBS; cells were counted and plated in T-75 flasks (4.5 million cells per flask) and grown to confluence. Confluent flasks were shaken at 250 rpm (37°C) in a rotary shaker for 2 hrs to disengage microglia and debris. Medium was replaced and cultures were shaken for two rounds of 24 hrs, at the end of which OPCs were harvested by centrifugation and resuspended in DMEM-HEPES +10%FBS. For differentiation studies, cells were plated on Poly-D-Lysine treated coverslips (5000 cells per coverslip) allowed to attach for 2 hrs before changing medium to differentiation medium (DMEM-HEPES 1%FBS+1x N2 supplement + 2 ng/ml Triiodothyronine). OPC enrichment was over 95% in all cell cultures (assayed through cell morphology and MBP expression upon differentiation). Drug treatments (Hedgehog inhibitor cyclopamine at 5 μM) were paired to drug vehicles (DMSO) and added to cultures to a 0.01% concentration. Recombinant octyl-modified Shh-N protein was used at 3.3 μg/ml (R&D Systems). OPC proliferation medium was a Neurobasal (Invitrogen, Life Technologies) supplemented with: 2mM L- glutamine, B27 supplement 2% (CAT# 17504–044, Gibco, Life Technologies), 10ng/mL de PDGFα (CAT#P3076–10 UG, Sigma), 10ng/mL bFGF (CAT#F5392 – 1UG, Sigma).

### Transfection with constitutively active SMO

OPCs were transiently transfected with an expression plasmid that encodes a myc-flagged constitutively active form Smo (W539L, SmoA1; [17] see [43] for details) using Amaxa-Nucleofector II (Lonza, Switzerland). Briefly, 10^6^ OPCs were recovered by trypsinization and resuspended in nucleofection solution along with 1ug of expression plasmid containing a flagged constitutively active Smo (SmoA1) (see [43] for details) and 1ug of GL-GFP (Invitrogen). Electroporated cells were recovered to with DMEM+10% FBS and plated on Poly-D-Lysine treated coverslips as described above.

### Immunofluorescence and imaging

PBS washed cells were fixed by a 5 minute incubation in 4%PFA + 4% sucrose in PBS (RT) or 20 min incubation in 100% methanol (-20° C) followed by 3 washes with PBS. Cells were permeabilized with PBS + 0.1% TX-100, washed twice with PBS, blocked with PBS + 5% BSA for 3 hrs and incubated with primary antibodies overnight (4°C, in humidified chamber), washed four times in PBS and incubated with corresponding secondary antibodies for 2 hrs (RT). DAPI and/or ToPro3 was used for nuclear staining. Coverslips were washed 4 times, and mounted on glass slides using Fluorescent Mounting Medium (DAKO #S3023, USA). Images were obtained at the advanced microscopy unit of the Faculty of Science using Zeiss confocal microscopes (Zeiss 510-Meta confocal microscope or Zeiss 710 confocal).

### BrdU Incorporation

OPCs were treated with a BrdU for 3 hrs (1ug/ml) in the presence or absence of PDGF (10ng/ml), washed and fixed for 20 minutes in 4% PFA, followed by two washes with PBS and incubated at 37°C with 2M HCL for 20 minutes. Coverslips were then rinsed three times with borate buffer (0.1M; pH 8.5) for 5 minutes. Subsequently, cultures were processed for immunofluorescence as described above using an anti-BrdU antibody

### Scholl analysis

Immunofluorescent anti-tubulin staining of OLGs served to assess process complexity as a measure of cell differentiation as assayed by Scholl analysis [44] using the ImageJ Sholl Analysis Plugin (v1.0) according to the developers’ instructions.

### Lactate dehydrogenase (LDH)

Cell culture supernatant (10 μl) was mixed with 140 μl freshly prepared LDH assay reagent to reach final concentrations of 821 nM Pyruvic acid, 214 nM nicotinamide adenine dinucleotide, 210,1 mM Sodium Chloride and 94,64 mM Tris (pH 7.2). The changes in absorbance were read kinetically at 340 nm for 20 min on Bioteck (Synergy 2).

### Statistical analysis

Drug effects on process extension or PC presence were analyzed by Kruskal-Wallis non-parametric test using a confidence level of 95% (α = 0.05).

## Supporting Information

S1 Checklist All procedures and data evaluation was certified. The present manuscript (PONE-D-15-13290) was revised and found in compliance que the NC3Rs ARRIVE Guidelines Checklist (included).
(PDF)

S1 Fig Constitutively active SmoA1-Flag proteins concentrate around γ-tubulin in isolated rat OPCs. OPCs transiently transfected using an Amaxa-Nucleofector II and associated nucleofection kit (Lonza, Switzerland). One million OPCs were nucleofected with 1ug of a constitutively active myc-flagged-Smo (SmoA1) (see [43] for details) and 1ug of GL-GFP (Invitrogen). Cells were recovered to with DMEM+10% FBS and plated on Poly-D-Lysine treated coverslips as described above. OPCs co-transfected with GL-GFP and SMO-Myc-flag plasmids were cultured for 24 hours, fixed and stained for γ-tubulin and myc-flagged proteins. Anti Myc-Flag antibodies marked GFP positive cells in areas in apposition to γ-tubulin staining. Bars: 5 μm.
(TIF)

S2 Fig Cyclopamine and Ciliobrevin 24 hr treatments do not increase the release of LDH in to the cell culture supernatant. OPCs were cultured under proliferative conditions in the presence or absence of Cyclopamine, Ciliobrevin or control solutions. Cell culture supernatant was assayed for LDH activity as a measure of membrane damage and cell death. No significant divergence was observed between control and treated cells. Cells subjected to 2% TritonX-100 were included as positive control.
(TIF)

S3 Fig Ciliobrevin does not reduce the number of OPCs detected. Proliferating OPCs (medium supplemented with PDGF-AA) were pretreated for 3 hrs with ciliobrevin (30 μM) and/or with proliferation medium, after which they received 3.3 μg/ml of recombinant Shh and were cultured for 24 hrs. Cells were stained with DAPI and counted in a double blind fashion in 30 random fields. Graph shows the number of cells per field ± s.e.m. *P< 0,01 (Kruskal Wallis test).
(TIF)

S4 Fig Paraformaldehyde fixation of differentiating OPCs acetylated tubulin labeling of PC by allowing the antibodies to react to a large proportion of the cell microtubules. Rat OPCs were washed with PBS and fixed for 20 minutes in a 4% paraformaldehyde solution (in PBS) two hrs after plating (0 hrs in differentiation medium) and for the following 24, 48 and 72 hrs. Coverslips were processed for immunofluorescence using antibodies directed against Acetylated Tubulin (green) and MBP (red), while nuclei were stained with ToPro3. Notice that the staining of a large proportion of cell microtubules obscures any PC immunoreactivity. Bars: 10 μm.
(TIF)

S1 Video PC markers localize to a single rod-shaped structure that project from the surface of Rat OPCs. The specific primary cilium markers glutamylated tubulin (green) and γ-tubulin (red) were detected by immunofluorescence after 48 hrs in differentiation medium. Confocal microscopy images were stacked and projected into a 3D model using ImageJ (NIH, Bethesda, MD, USA) with minimal modifications (brightness-contrast). The video was created from the 3D projection generated.
(GIF)
