# Supplementary figures and images for: Shh Signaling through the Primary Cilium Modulates Rat Oligodendrocyte Differentiation
Source: PLoS One. 2015 Jul 28;10(7):e0133567. doi: 10.1371/journal.pone.0133567 (PMC4517900; doi:10.1371/journal.pone.0133567)

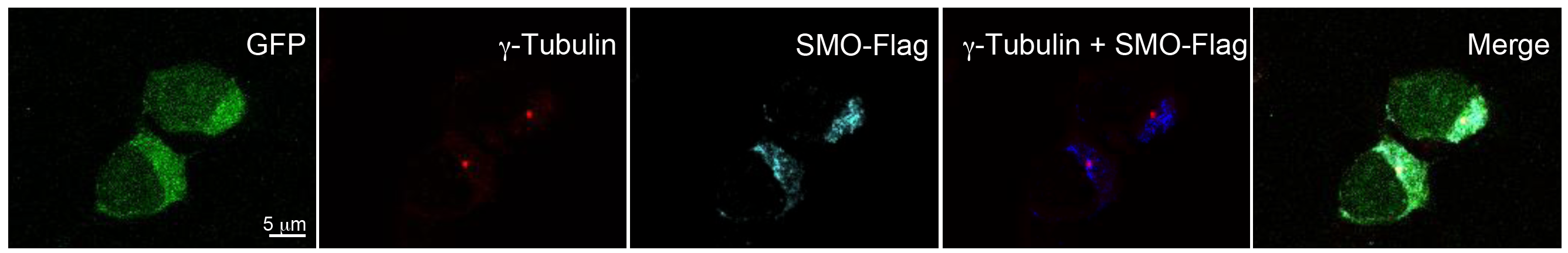

Supplement: S1 Fig — OPCs transiently transfected using an Amaxa-Nucleofector II and associated nucleofection kit (Lonza, Switzerland). One million OPCs were nucleofected with 1ug of a constitutively active myc-flagged-Smo (SmoA1) (see [43] for details) and 1ug of GL-GFP (Invitrogen). Cells were recovered to with DMEM+10% FBS and plated on Poly-D-Lysine treated coverslips as described above. OPCs co-transfected with GL-GFP and SMO-Myc-flag plasmids were cultured for 24 hours, fixed and stained for γ-tubulin and myc-flagged proteins. Anti Myc-Flag antibodies marked GFP positive cells in areas in apposition to γ-tubulin staining. Bars: 5 μm. (TIF) [file pone.0133567.s002.tif]

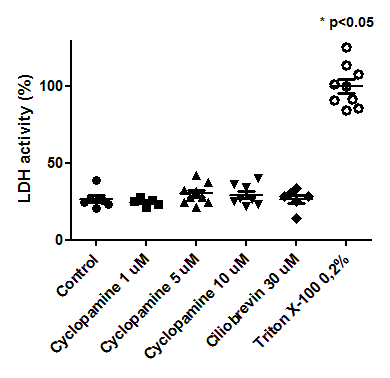

Supplement: S2 Fig — OPCs were cultured under proliferative conditions in the presence or absence of Cyclopamine, Ciliobrevin or control solutions. Cell culture supernatant was assayed for LDH activity as a measure of membrane damage and cell death. No significant divergence was observed between control and treated cells. Cells subjected to 2% TritonX-100 were included as positive control. (TIF) [file pone.0133567.s003.tif]

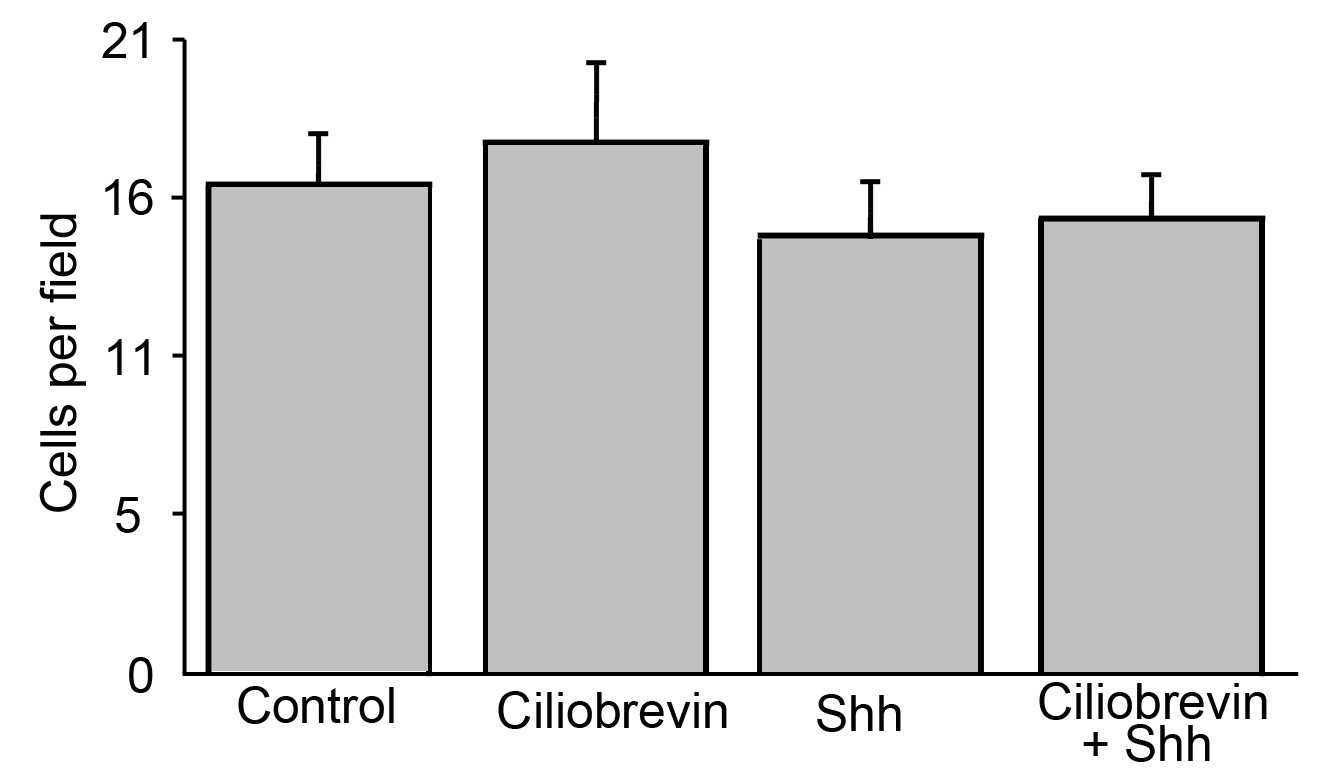

Supplement: S3 Fig — Proliferating OPCs (medium supplemented with PDGF-AA) were pretreated for 3 hrs with ciliobrevin (30 μM) and/or with proliferation medium, after which they received 3.3 μg/ml of recombinant Shh and were cultured for 24 hrs. Cells were stained with DAPI and counted in a double blind fashion in 30 random fields. Graph shows the number of cells per field ± s.e.m. *P< 0,01 (Kruskal Wallis test). (TIF) [file pone.0133567.s004.tif]

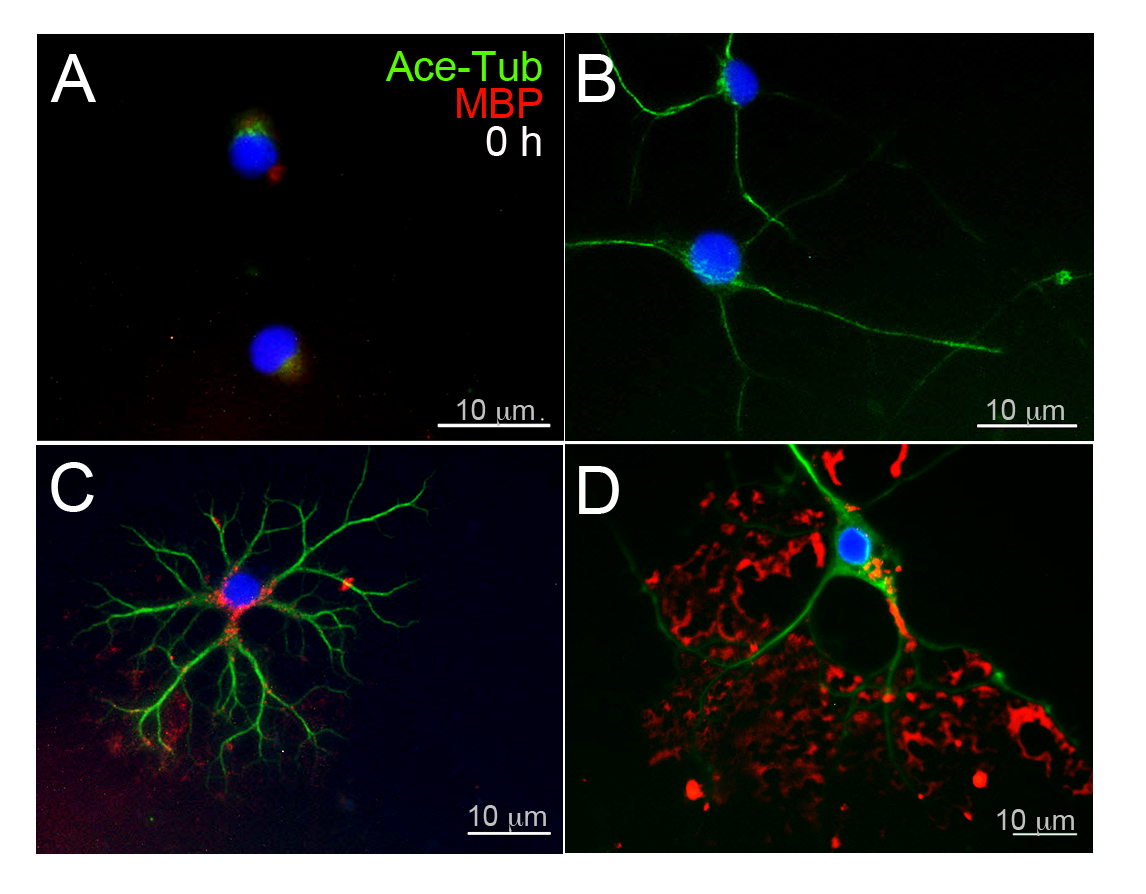

Supplement: S4 Fig — Rat OPCs were washed with PBS and fixed for 20 minutes in a 4% paraformaldehyde solution (in PBS) two hrs after plating (0 hrs in differentiation medium) and for the following 24, 48 and 72 hrs. Coverslips were processed for immunofluorescence using antibodies directed against Acetylated Tubulin (green) and MBP (red), while nuclei were stained with ToPro3. Notice that the staining of a large proportion of cell microtubules obscures any PC immunoreactivity. Bars: 10 μm. (TIF) [file pone.0133567.s005.tif]

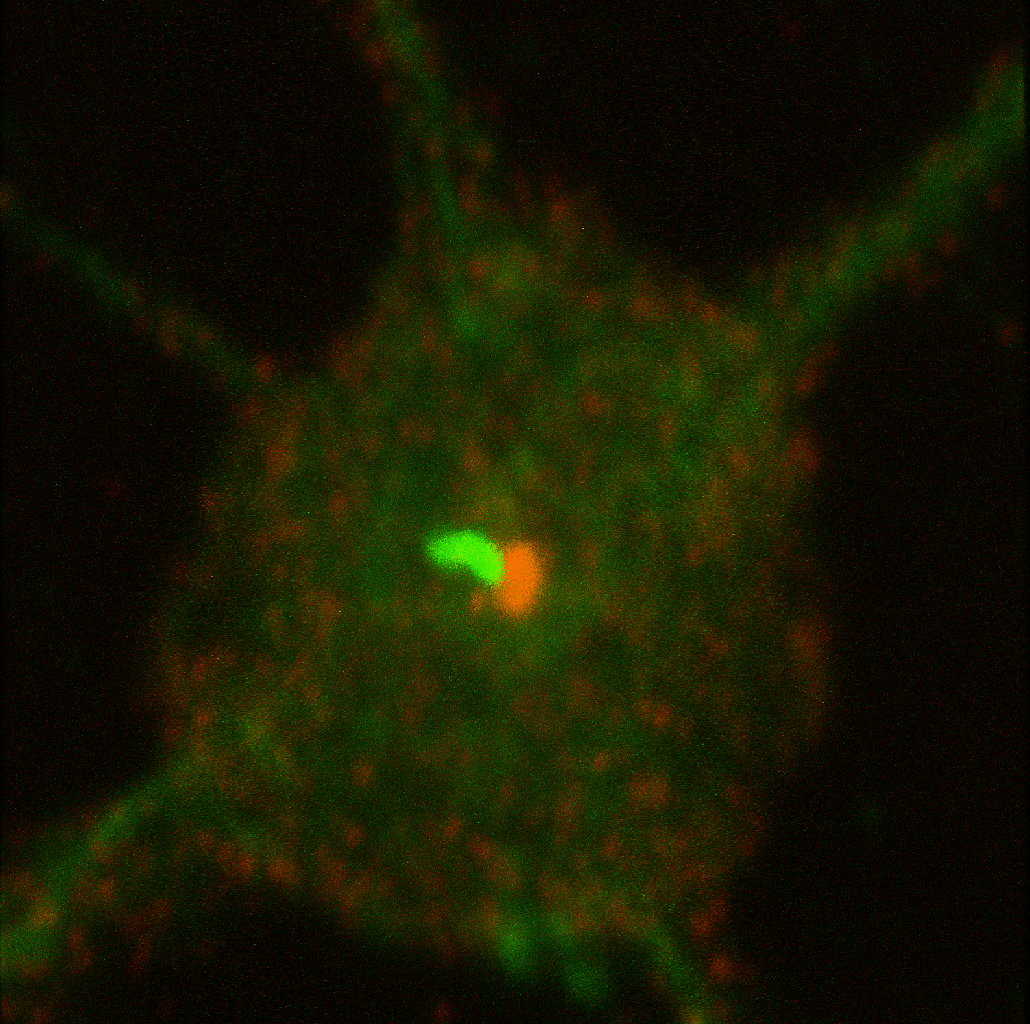

Supplement: S1 Video — The specific primary cilium markers glutamylated tubulin (green) and γ-tubulin (red) were detected by immunofluorescence after 48 hrs in differentiation medium. Confocal microscopy images were stacked and projected into a 3D model using ImageJ (NIH, Bethesda, MD, USA) with minimal modifications (brightness-contrast). The video was created from the 3D projection generated. (GIF) [file pone.0133567.s006.gif]
